# Can Anthropometry and Body Composition Explain Physical Fitness Levels in School-Aged Children?

**DOI:** 10.3390/children8060460

**Published:** 2021-05-31

**Authors:** Chih-Yu Hsu, Liang-Sien Chen, I-Jen Chang, Wei-Ching Fang, Sun-Weng Huang, Rong-Ho Lin, Steve Wen-Neng Ueng, Hai-Hua Chuang

**Affiliations:** 1 Department of Family Medicine, Linkou and Taipei Branches, Chang Gung Memorial Hospital, Taoyuan 33305, Taiwan; b85079@gmail.com (C.-Y.H.); m7090@cgmh.org.tw (L.-S.C.); Drchangijen@gmail.com (I.-J.C.); winds75526@gmail.com (W.-C.F.); 2 Department of Industrial Engineering and Management, National Taipei University of Technology, Taipei 10608, Taiwan; t107379004@ntut.org.tw (S.-W.H.); rhlin@mail.ntut.edu.tw (R.-H.L.); 3 Graduate Institute of Urban Planning, College of Public Affairs, National Taipei University, New Taipei City 23741, Taiwan; 4 College of Medicine, Chang Gung University, Taoyuan 33302, Taiwan; wenneng@adm.cgmh.org.tw; 5 Department of Orthopedic Surgery, Linkou Main Branch, Chang Gung Memorial Hospital, Taoyuan 33305, Taiwan; 6 Obesity Institute, Geisinger, Danville, PA 17837, USA

**Keywords:** anthropometry, body composition, children, epidemiography, physical fitness

## Abstract

Physical fitness (PF) is closely related to various health outcomes and quality of life among children. However, the associations between anthropometry, body composition (BC), and PF are not fully elucidated. This cross-sectional study aimed to investigate the associations between demographic metrics (age, sex), anthropometric measures (body mass index z-score (BMI z-score) waist/height ratio (WHtR)), BC parameters (body-fat percentage (BF%), muscle weight), and PF levels (800-m run, sit-and-reach, 1-min sit-ups, standing long jump) in school-aged children. Continuous variables were dichotomized by median splits. The results of 180 girls and 180 boys (mean age: 10.0 ± 0.7 years; mean BMI z-score: 0.366 ± 1.216) were analyzed. Multivariable linear regressions revealed that BF% (regression coefficient (B) = 3.4, 95% confidence interval (CI) = 2.5–4.3) was independently correlated with the 800-m run. Sex (B = 4.6, 95% CI = 3.0–6.3), age (B = 3.1, 95% CI = 1.9–4.3), and BMI z-score (B = −0.7, 95% CI = −1.4–−0.1) were independently related to sit-and-reach. Age (B = 3.3, 95% CI = 2.0–4.7), BF% (B = −0.3, 95% CI = −0.4–−0.2), and muscle weight (B = 0.7, 95% CI = 0.2–1.2) were independently associated with 1-min sit-ups. In addition to demography, anthropometry and BC provided additional information concerning some PF levels in school-aged children. Weight management and PF promotion should be addressed simultaneously in terms of preventive medicine and health promotion for children.

## 1. Introduction

Physical fitness (PF) is defined as one’s capability to work effectively, enjoy leisure time, be healthy, resist hypokinetic diseases, and meet emergencies [1,2]. PF also displays the body’s ability to function efficiently. A substantial volume of research has been conducted in adults on the relationships between PF and various health outcomes [3]. The literature suggests that a lack of physical activity (PA) or poor PF is associated with a wide range of chronic diseases [4]. PF is an important predictor for adiposity, bone health, premature mortality, diabetes, cardiovascular diseases, metabolic syndrome, and mental health [3,5,6,7,8].

Among all the PF metrics, cardiorespiratory and muscular fitness seem to be the two most influential parameters for disease and health [9]. Low cardiorespiratory fitness is associated with metabolic syndrome, diabetes, and an increased prevalence of cardiovascular disease [7,10]. A recent review elucidates that good cardiorespiratory fitness mitigates stroke hazard in both sexes, and the effect is comparable to well-established stroke risk factors such as blood pressure, obesity, and smoking [4,11]. Muscular strength is a commonly accepted parameter for cardiometabolic risk, sarcopenia, functional disabilities, and frailty connected to all-cause mortality [12,13,14].

PF is also related to mental health. Previous studies have observed an association between the PF component and cognitive development [8,9,15]. Evidence supports that reduced cardiorespiratory fitness and muscular strength are linked to lower levels of mental well-being, higher levels of depressive symptoms and stress-related exhaustion, and increased health-risk behavior such as suicidal attempts [9]. Silverman and Deuster have proposed two possible mechanisms to explain how PF can buffer against stress-related diseases. The first one is the “blunting effects” of PF on stress responses from the hypothalamic–pituitary–adrenal axis or the sympathetic nervous system. The other one is that improving PF may minimize excessive systemic inflammation. Moreover, PF enhances the neural plasticity and the expression of growth factor, and therefore improves an individual’s mood and cognition [16].

Similar observations have been reported among children. Children with suboptimal PF are at higher risk of pediatric obesity, type 2 diabetes, hypercholesterolemia, hypertension, metabolic syndrome, and cardiovascular diseases [17,18,19]. Poor childhood PF is linked to increased all-cause mortality in adulthood [5,20]. A low level of PF is associated with excessive inflammatory cytokine production [21], with promoting adipose tissue aggregate [22], disrupting platelet function [23], and increasing insulin resistance [24,25]. Moreover, one cross-sectional study suggests that PF plays a crucial role and may ameliorate the negative influence of obesity on children’s academic performance [15,26].

Notably, the effects of PF on health outcomes are independent of weight status. Individuals with poor PF, even normal-weighted, have higher mortality than individuals with optimal PF status [27]. Obesity (defined with anthropometric measures), suboptimal body composition (BC), and poor PF have various comorbidities in common, such as metabolic alterations, cardiovascular risks, and mental–cognitive impairment. However, most studies have focused on the medical impacts of each of the three, and the relationships between anthropometry, BC, and PF have not been studied as extensively. Mendoza-Muñoz et al. investigated the influence of BC on PF in adolescents and reported that overweight and obese adolescents had lower levels of PF performance [28]. Ortega, F.B., et al. and Hussey, J., et al. demonstrated that cardiorespiratory fitness was negatively correlated with body mass index (BMI), waist circumference and abdominal adiposity in children [5,29]. However, connections between anthropometric measures, BC, and PF in school-aged children had not yet been studied and elucidated thoroughly.

We hypothesized that not only anthropometry but also BC was associated with PF performance among children. The aim of this study was to investigate the associations between body mass index (BMI) z-score, waist/height ratio (WHtR), body-fat percentage (BF%), muscle weight (MW), 800-m run, sit-and-reach, 1-min sit-ups, and standing long jump in a sample of elementary students in Taiwan.

## 2. Materials and Methods

### 2.1. Study Design and Subjects

The study was quantitative and cross-sectional. Data were collected between 2013 and 2016 from a school-based health promotion project conducted by the Chang Gung Memorial Hospital, Linkou Main Branch, Taoyuan, Taiwan for several elementary schools in Northern Taiwan. The details of the project have been reported elsewhere [30]. Anonymous data was retrieved and analyzed in 2020. All sensitive pieces of information that could be linked to a specific person were deidentified. Demographic data (age, sex), anthropometric measures (BMI z-score, WHtR), BC measures (BF, MW), and PF outcomes (cardiorespiratory fitness (800-m run), flexibility (sit-and-reach), speed/agility (1-min sit-ups), lower body power (long-jump)) were analyzed. This study was approved by the Institutional Review Board of our hospital (101-4158A3). Written informed consent was obtained from all participants and their parents.

### 2.2. Anthropometric Measures

Anthropometric measures were obtained collaboratively by school teachers, nurses, and investigators in elementary schools. Each student was asked to take off his or her shoes before body height and weight were measured according to standard protocols [31]. Body height was assessed to the nearest 0.1 cm by using a wall-mounted stadiometer with head held in horizontal plane [32]. Body weight was measured to the nearest 0.1 kg by using a metric balance scale [33]. BMI was calculated by dividing the weight (kg) by the height squared (m^2^) [31]. To make weight status comparable across children at different ages, BMI z-scores were calculated based on sex and age in months according to the United States Centers for Disease Control and Prevention 2000 growth charts [34]. Waist circumference (cm) was measured as the circumference in the horizontal plane midway between the lowest ribs and the iliac crest, and it was further divided by the height (cm) to calculate WHtR [35,36].

### 2.3. Body Composition Parameters

The detailed BC measurement protocol has been described elsewhere [30]. Briefly, BF% and MW were automatically obtained using bioelectrical impedance analysis (X-scan model, Jawon Medical, Co., Ltd., Seoul, Korea). This segmental multi-frequency bioelectrical impedance analysis module can estimate not only fat-free mass/fat mass but also muscle weight, intracellular water weight and extracellular water weight across limbs and trunks [37]. We chose MW rather than fat-free mass as our parameter of interest since fat-free mass is more complex in its components, including not only muscles but also bone mineral, extracellular water, intracellular water, and visceral protein [37]. Jensen, B., et al. indicated that skeletal muscle mass accounted for 45% to 49% of fat-free mass across different sexes [38].

### 2.4. Physical Fitness Levels

PF levels were measured by four exercise tests, including 800-m run, sit-and-reach, 1-min sit-ups, and standing long jump. Cardiorespiratory fitness (endurance run) was assessed by 800-m run (sec), defined as the time required to sprint 800-m run [39,40]. Flexibility was evaluated using sit-and-reach (cm), defined by the maximum distance between fingertips and feet reached as the participants slide their hands forward as far as possible towards their feet without bending the hamstring, and maintain this maximum position for at least two seconds [41]. Speed/agility was measured by 1-min sit-ups (times), defined as the maximum number of correct sit-ups achieved in one minute [42]. Lower body power was evaluated using standing long jump, defined as the distance between the starting line and the heel of the closest foot [43]. All PF tests were performed following the methods reported by the Health Promotion Administration, Ministry of Health and Welfare in Taiwan. Standard and corrected stopwatches were used to measure time. Professional physical educators were enrolled to conduct the tests and document the results [44].

### 2.5. Statistical Analysis

The sample size was calculated using a priori calculation (correlation test, coefficient of determination ρ2 = 0.11, two-tailed α = 0.05, power = 0.85; sample size for each age–sex subgroup = 60), as reported in a previous study of BF% and standing long jump in 9-year-old boys [43]. For boys and girls aged 9–11 years old (six age–sex subgroups), the sample size was estimated to be 360.

Most of the distributions of the variables were non-normal, assessed by using the Kolmogorov–Smirnov test. Therefore, non-normally distributed continuous variables were transformed using a two-step approach: the fractional rank and inverse–normal transformation [45]. Thereafter, continuous variables were expressed as means and standard deviations, and categorical variables were expressed as numbers with percentages. Student *t*-tests were used to compare continuous variables, and chi-square tests were used to compare categorical variables in different subgroups, as appropriate. Pearson’s correlation test was used to investigate the relationships between continuous variables, whereas Spearman’s correlation test was used to analyze associations between continuous variables and categorical variables. The variables with a significance level of *p* < 0.05 in univariate analyses were further assessed using multivariable linear regression models with the forward selection for examining independent variables.

SPSS software (version 25; International Business Machines Corp., Armonk, NY, USA) and G*Power software (version 3.1.9.2; University of Kiel, Kiel, Germany) were used for the statistical analysis. A two-tailed *p*-value lower than 0.05 was considered statistically significant.

## 3. Results

### 3.1. Demographic and Clinical Characteristics of the Overall Cohort and Two Subgroups Stratified by Sex

Table 1 displayed demographic and clinical characteristics of the overall cohort and two subgroups stratifies by sex. A total of 360 Taiwanese children from Han ancestry (180 (50.0%) girls and 180 (50.0%) boys); mean age: 10.0 ± 0.7 years; mean BMI z-score: 0.366 ± 1.216) were analyzed.

In the overall cohort, the boys had a significantly higher BMI z-score, higher WHtR, lower BF%, higher MW, lower time in the 800-m run, lower distance of sit-and-reach, higher number of 1-min sit-ups, and higher distance of standing long jump compared to those of the girls. The boys and girls were comparable in age.

### 3.2. Associations of Physical Fitness Levels and Variables of Interest in the Overall Cohort

Table 2 displayed associations of PF and variables of interest in the overall cohort. The 800-m run was positively associated with female sex, BMI z-score, WHtR, BF%, and MW. Sit-and-reach was positively correlated with female sex and age, and inversely related to BMI z-score and WHtR. The 1-min sit-ups score was positively related to age and inversely associated with BMI z-score, WHtR, and BF%. Standing long jump was positively associated with age and MW, and inversely correlated with female sex, WHtR, and BF%.

### 3.3. Variables Independently Associated with Physical Fitness Levels in the Overall Cohort Using Logistic Regression Models

Table 3 displayed variables independently associated with PF in the overall cohort. Using univariate linear regression models, we determined that girls, BMI z-score, WHtR, BF%, and MW were significant variables of the 800-m run. However, only the BF% (regression coefficient (B) = 3.4, 95% confidence interval (CI) = 2.5–4.3, *p* < 0.001) was independently correlated with the 800-m run using multivariable linear regression models.

Female sex, age, BMI z-score, and WHtR were significant variables of sit-and-reach. Using multivariable analysis, girls (B = 4.6, 95% CI = 3.0–6.3, *p* < 0.001), age (B = 3.1, 95% CI = 1.9–4.3, *p* < 0.001), and BMI z-score (B = −0.7, 95% CI = −1.4–−0.1, *p* = 0.03) were still significantly and independently related to the sit-and-reach.

Female sex, age, BMI z-score, WHtR, and BF% were significant factors of 1-min sit-ups. Furthermore, age (B = 3.3, 95% CI = 2.0–4.7, *p* < 0.001) and BF% (B = −0.3, 95% CI = −0.4–−0.2, *p* < 0.001) were still significant variables of 1-min sit-and-stand-ups using multivariable analysis.

Female sex, age, WHtR, BF%, and MW were significant factors of the standing long jump. Their associations with the standing long jump remained significant using multivariable analysis: age (B = 14.1, 95% CI = 9.9–18.3, *p* < 0.001), BF% (B = −1.1, 95% CI = −1.4–1.2, *p* < 0.001), and MW (B = 0.7, 95% CI = 0.2–1.2, *p* = 0.01).

## 4. Discussion

The prevalence of pediatric obesity has been increasing alarmingly worldwide in the past 20 years [46,47,48]. It is generally believed that a decrease in PA and excessive calorie consumption are the two most important factors responsible for this obesity pandemic [49,50]. The detrimental effects of obesity can result in a wide range of negative impacts on health and quality of life [51]. The literature has elucidated various comorbidities of pediatric obesity, including early sexual maturation, polycystic ovary syndrome, nonalcoholic fatty liver disease, obstructive sleep apnea, asthma, left ventricular hypertrophy, hypertension, lower extremity malalignment and joint pain, acanthosis nigricans, higher risk of idiopathic intracranial hypertension, poor self-esteem, anxiety, and depression [51].

On the other hand, a decrease in PF performance has been observed in many developing and developed countries [50]. Young populations are becoming more obese and less fit at the same time, and some research has suggested a linkage between the changes in PF and weight status [50,52,53]. Overweight and obese adolescents had inferior PF compared to their normal-weight peers [28]. Children with higher cardiorespiratory fitness had less total and abdominal adiposity [5,28]. Tomkinson et al. demonstrated that the variability in fatness was accounted for about 20% of the variability in running performance [54]. A cross-sectional study also showed that fatness attributed to the decline of PF by 29–61%, more than other factors such as PA did [52].

In our results, and consistent with the literature, the PF performance of children was associated with their demographic variables [55]. Older children had better flexibility, speed/agility, and lower body power. Children grow up with their height, weight, lean mass, fat tissue, and organs increasing in size [56,57]. These changes are the results of cellular hyperplasia, hypertrophy, and intercellular accretion, enhancing the functional utility of skeletal muscles [58]. Moreover, children develop better motor performance with a better integration of their central nervous system and skeletal–muscular system [59]. Previous investigations report that older age is associated with higher running speed from increased stride length, frequency, and neuromuscular coordination [60,61].

Interesting sex differences were observed. Boys were higher in z-BMI and WHtR, consistent with the well-documented higher prevalence of overweight and obesity in boys in Taiwan [62,63]. Girls were higher in BF% and lower in MW, also consistent with the literature [64,65]. After adjusting for other confounders, girls were better with flexibility. Previous studies show that girls have more total and subcutaneous fat, more connective tissue, and less paraspinous musculature compared to boys [64,66]. This difference could be explained by the difference in sex hormone levels between prepubertal children [67]. Boys have a greater capacity of skeletal muscle because of a higher level of testosterone [57,58]. In comparison, girls have a higher BF% and lower percentage of muscle mass because of a higher amount of estrogen, which contributes to lower tissue density and better flexibility [66]. The divergence of sexual hormone level and BC causes distinct flexibility performance between different sexes [68,69].

As expected and consistent with some previous research [29,70,71], our data demonstrated connections between weight status, BC, and PF. In general, higher BMI-z score and BF% were linked with lower levels of PF.

First, higher z-BMI was associated with worse flexibility. Larger trunk mass increased mechanical work and moment of inertia. As a result, there was higher propulsion and extra load during dynamic activities [70]. Casonatto et al. indicated that abdominal obesity might affect the lower back and hamstring flexibility and hamper the trunk to the extreme reach position [72].

Second, excessive BF% was related to poorer muscle endurance, cardiorespiratory fitness, and lower-body power. Similar findings have been reported by other investigators [28,73,74,75]. One systematic review from the European Childhood Obesity Group suggests that insulin resistance causes mitochondrial dysfunction in the skeletal muscle, which leads to muscle fatigability and delayed post-effort muscle recovery [76]. Another systematic review shows the negative effects of childhood obesity on spirometric variables, namely reductions in forced expiratory volume in one second, forced vital capacity, and forced expiratory volume in one second/forced vital capacity ratio [77,78]. An increase in adipose tissue directly negatively impacts one’s pulmonary function by reducing functional residual capacity, expiratory reserve volume, and residual volume [79,80]. Previous studies have reported that children with obesity perform less well in weight-bearing activities such as jumping and running [53,75,81]. Children with higher BMI usually have higher muscle mass, which increases absolute muscle strength; however, relative muscle strength is the key component of muscle function for daily activities. Excessive BF% increases inert load, which impacts negatively on relative muscle strength and results in poorer lower-body power [75].

Third, children with higher muscle weight had better lower-body power. There are two types of skeletal muscle, type I (red fibers) and type II (white fibers). Type II fibers have higher force, power, and speed. Most one-burst moves, such as the long jump or badminton smash, are largely contributed by type II fibers due to their higher contractility and fast reactions. On the other hand, type I fibers are slow movers but have better ability in utilizing oxygen and higher endurance [82]. Gaining muscle weight is largely related to type II fiber rather than type I fibers [83]. Our results were consistent with the physiology, showing that muscle weight was significant in relation to lower-body explosive power, but not muscle endurance.

The main contribution of this study was to provide scientific evidence on how anthropometry and BC may affect the PF performance, in addition to age and sex. The study had some limitations. First, all the participants were Han and confined to age of 9–11 years, which might limit the generalizability of this study to other populations. Second, the information on pubertal status was not collected, which might have been an important confounder on weight status, BC, and PF. Third, BC was measured by BIA; BIA modules are less costly and more available, but less accurate than dual energy X-ray absorptiometry modules. Also, the study was cross-sectional and therefore unable to conduct causation in predicting the future performance. Further studies with a prospective design will be of interest.

## 5. Conclusions

Overall, younger age, higher BMI z-score, and higher BF% were associated with poorer PF performance among elementary school students; girls had better flexibility. Our study concluded that other than demographic variables, anthropometric measures and BC parameters provided additional information in concerning PF levels in school-aged children. PF testing should be considered in addition to clinical measures to understand children’s health more comprehensively. Health promotion for children should focus not only on anthropometry but also BC and PF.

## Figures and Tables

**Table 1:** Participant characteristics of the overall cohort as well as two subgroups stratified by sex.

Variables	Overall	Boys	Girls	*p*-Value ^a^
Participants	*n* = 360	*n* = 180	*n* = 180	
Demographic measures				
Age (years)	10.0 ± 0.6	10.0 ± 0.7	10.0 ± 0.6	0.72
Anthropometric measures				
Body mass index z-score	0.366 ± 1.216	0.543 ± 1.269	0.190 ± 1.137	0.01
Waist/height ratio	0.46 ± 0.06	0.47 ± 0.06	0.45 ± 0.06	<0.001
Body composition measures				
Body-fat percentage	16.9 ± 7.6	14.1 ± 8.0	19.5 ± 6.1	<0.001
Muscle weight (kg)	29.0 ± 6.1	30.7 ± 5.6	27.5 ± 6.2	<0.001
Physical fitness levels				
800-m run (sec)	306.9 ± 69.4	297.1 ± 74.2	316.7 ± 63.0	0.01
Sit-and-reach (cm)	25.6 ± 8.4	23.1 ± 8.1	28.1 ± 7.9	<0.001
1-min sit-ups (time)	27.8 ± 8.8	28.7 ± 9.6	26.9 ± 7.9	0.049
Standing long jump (cm)	137.1 ± 25.6	141.1 ± 27.0	133.1 ± 23.5	0.003

Data are summarized as means ± standard deviations. ^a^ Data were compared using the Student *t*-test.

**Table 2:** Associations ^a^ of dichotomized variables of interest.

Predictors	Female Sex ^a^	Age ^b^	Body Mass Index z-Score ^b^	Waist/Height Ratio ^b^	Body-Fat Percentage ^b^	Muscle Weight ^b^	800-m Run ^b^	Sit-and-Reach ^b^	1-min Sit-Ups ^b^	Standing Long Jump ^b^
Female sex	–									
Age	0.02 (0.72)	–								
Body mass index z-score	−0.14 (0.01 *)	−0.03 (0.63)	–							
Waist/height ratio	−0.19 (<0.001 *)	−0.05 (0.37)	0.80 (<0.001 *)	–						
Body-fat percentage	0.36 (<0.001 *)	0.03 (0.57)	0.69 (<0.001 *)	0.62 (<0.001 *)	–					
Muscle weight	−0.24 (<0.001 *)	0.44 (<0.001 *)	0.68 (<0.001 *)	0.43 (<0.001 *)	0.43 (<0.001 *)	–				
800-m run	0.15 (0.004 *)	−0.04 (0.42)	0.28 (<0.001 *)	0.27 (<0.001 *)	0.37 (<0.001 *)	0.11 (0.04 *)	–			
Sit-and-reach	0.32 (<0.001 *)	0.26 (<0.001 *)	−0.15 (0.004 *)	−0.15 (0.01 *)	0.02 (0.69)	−0.07 (0.22)	−0.04 (0.51)	–		
1-min sit-ups	−0.10 (0.06)	0.22 (<0.001 *)	−0.14 (0.01 *)	−0.18 (<0.001 *)	−0.28 (<0.001 *)	0.01 (0.92)	−0.48 (<0.001 *)	0.08 (0.11)	–	
Standing long jump	−0.15 (0.004 *)	0.42 (<0.001 *)	−0.10 (0.06)	−0.11 (0.04 *)	−0.24 (<0.001 *)	0.18 (0.001 *)	−0.39 (<0.001 *)	0.27 (<0.001 *)	0.44 (<0.001 *)	–

Data are summarized as Spearman correlation coefficients (*p*-values). ^a^ Data were analyzed using the Spearman correlation test. ^b^ Data were analyzed using the Pearson correlation test. * *p*-value < 0.05.

**Table 3:** Univariate and multivariable regression models of physical fitness levels in the overall cohort.

Predictors	B (95%CI)	*p*-Value ^a^	B (95%CI)	*p*-Value ^a^
	Univariate Model		Multivariate Model	
800-m run				
Female sex	19.6 (5.3–33.9)	0.01		NS
Age	−4.5 (−15.5–6.5)	0.42		NI
Body mass index z-score	15.9 (10.2–21.6)	<0.001		NS
Waist/height ratio	290.5 (180.7–400.2)	<0.001		NS
Body-fat percentage	3.3 (2.4–4.2)	<0.001	3.4 (2.5–4.3)	<0.001
Muscle weight	1.3 (0.1–2.5)	0.04		NS
Sit-and-reach				
Female sex	5.0 (3.4–6.7)	<0.001	4.6 (3.0–6.3)	<0.001
Age	3.3 (2.0–4.6)	<0.001	3.1 (1.9–4.3)	<0.001
Body mass index z-score	−1.1 (−1.8–−0.3)	0.004	−0.7 (−1.4–−0.1)	0.03
Waist/height ratio	−19.0 (−32.5–−5.5)	0.01		NS
Body-fat percentage	0.02 (−0.10–0.14)	0.69		NI
Muscle weight	−0.1 (−0.2–0.1)	0.22		NI
1-min sit-ups				
Female sex	−1.8 (−3.7–−0.1)	0.049		NS
Age	2.9 (1.6–4.3)	<0.001	3.3 (2.0–4.7)	<0.001
Body mass index z-score	−1.0 (−1.8–−0.3)	0.01		NS
Waist/height ratio	−24.8 (−38.9–−10.7)	0.001		NS
Body-fat percentage	−0.3 (−0.4–−0.2)	<0.001	−0.3 (−0.4–−0.2)	<0.001
Muscle weight	0.1 (−0.1–0.2)	0.92		NI
Standing long jump				
Female sex	−8.0 (−13.2–−2.7)	0.003		NS
Age	16.3 (12.6–20.0)	<0.001	14.1 (9.9–18.3)	<0.001
Body mass index z-score	−2.1 (−4.3–0.1)	0.06		NS
Waist/height ratio	−42.5 (−83.8–−1.1)	0.04		NS
Body-fat percentage	−0.8 (−1.2–−0.5)	<0.001	−1.1 (−1.4–−0.7)	<0.001
Muscle weight	0.8 (0.3–1.2)	0.001	0.7 (0.2–1.2)	0.01

Abbreviations: B: regression coefficient; CI: confidence interval; NI: not included; NS: not significant. ^a^ Data were compared using linear regression models.

## Data Availability

Data available on request due to privacy/ethical restrictions.
